# Introgression affects *Salmo trutta* juvenile life‐history traits generations after stocking with non‐native strains

**DOI:** 10.1111/eva.13725

**Published:** 2024-07-02

**Authors:** Dorte Bekkevold, Francois Besnier, Thomas Frank‐Gopolos, Einar E. Nielsen, Kevin A. Glover

**Affiliations:** ^1^ National Institute of Aquatic Resources Technical University of Denmark Silkeborg Denmark; ^2^ Institute of Marine Research Bergen Norway

**Keywords:** admixture, common garden, domestication, hybrid, interaction, reaction norm, salmonid, temperature

## Abstract

Introgression of non‐native conspecifics changes the genetic composition of wild populations, potentially leading to loss of local adaptations and fitness declines. However, long‐term data from wild populations are still relatively few. Here, we studied the effects of introgression in a Danish brown trout (*Salmo trutta*, L.) population, subjected to intensive stocking with domesticated hatchery fish of non‐native origin. We used wild‐caught genetically wild and admixed trout as well as fish from the partly domesticated hatchery strain used for stocking the river up until ~15 years prior to this study, to produce 22 families varying in hatchery/wild admixture. Following a replicated common‐garden experiment conducted in fish tanks from first feeding through 23 weeks at 7, 12, and 16°C, we observed a significant positive relationship between family admixture and fish size upon termination, an effect observed through all levels of admixture. Furthermore, the admixture effect was most distinct at the higher rearing temperatures. Although the hatchery strain used for stocking had been in culture for ~7 generations, it had not been deliberately selected for increased growth. These data thus demonstrate: (i) that growth had increased in the hatchery strain even in the absence of deliberate directional selection for this trait, (ii) that the increasing effect of admixture by temperature could represent inadvertent selection for performance in the hatchery strain at higher temperatures, and most significantly, (iii) that despite undergoing up to five generations of natural selection in the admixed wild population, the genetically increased growth potential was still detectable and thus persistent. Our findings suggest that altered growth patterns and potentially their cascading effects are of importance to the severity of hatchery/wild introgression, especially under changing‐climate scenarios and are of general significance to conservation practitioners seeking to evaluate long‐term effects of intra‐specific hybridization including under recovery.

## 1 INTRODUCTION

Preservation of the evolutionary integrity of wild populations is a main topic in conservation and in this context, a main concern is the alteration of genetic variation caused by intentional or accidental introduction of cultured, or even domesticated, individuals into natural habitats (Laikre et al., 2010). Populations reared in captivity are subject to drivers that alter their genetic profiles making them diverge from their wild populations of origin. This is due to the combined effects of founder effects, genetic drift and changes in selection pressures (Frankham, 2008). Consequently, intraspecific hybridization and introgression (defined here as the transfer of gene variants of non‐native origin into a population's gene pool) is expected to lead to disruption of co‐adapted gene complexes and outbreeding depression that may reduce fitness in wild populations (Frankham et al., 2011).

In fishes, captive conditions typically differ from natural habitats by providing absence of predators, an abundance of high‐energy food, high rearing densities and environmental regimes optimized for growth (Hindar et al., 1991). Fish breeding programs are commonly applied to improve traits of commercial interest, such as growth, but even without directed selection, heritable changes can occur within a few generations of captivity (Araki et al., 2007, 2008; Berejikian & Ford, 2004; Christie et al., 2012; Frankham, 2008; Fraser, 2008). In salmonids, millions of cultured fish find their way into natural systems every year, either through farm escapes (Diserud et al., 2019; Glover et al., 2019) or by deliberate releases aimed at supplementing local populations for conservation and fisheries purposes (Heard, 1995; Kostow, 2009). Upon release, cultured fish and their offspring may affect wild populations through a range of ecological and genetic interactions, where interbreeding between wild and cultured fish is common.

A substantial body of literature has documented that hybridisation and introgression leads to altered genetic profiles in wild populations (e.g. Glover et al., 2012; Hansen et al., 2009; Lamaze et al., 2012; Leitwein et al., 2021; Marie et al., 2010). Hybrids between fish of wild and cultured origin tend to display intermediate phenotypes, suggestive of additive genetic effects on polygenic traits (e.g. Bryden et al., 2004; Einum & Fleming, 1997; Harvey et al., 2016; McGinnity et al., 1997, Tymchuk et al., 2006; but also see Roberge et al., 2008). Repeated backcrossing into wild populations is suggested to result in a gradual dilution of non‐native phenotypes (McGinnity et al., 2003; Tymchuk et al., 2006, 2007), and in some cases, a return to wild phenotypes may be attained after few generations of backcrossing (Vandersteen et al., 2012). However, with the exception of recently emerging data from Atlantic salmon (*S. salar* L.) studies (Besnier et al., 2022; Bolstad et al., 2021), knowledge about the long‐term effects of introgression from non‐native conspecifics on phenotypic traits in wild populations is still quite scarce (Christie et al., 2014).

The brown trout is an ecologically highly variable species, exhibiting substantial population sub‐structure throughout its distribution (Jonsson & Jonsson, 2011). It is a popular target for recreational fisheries and has therefore been extensively cultured with hatchery production primarily aimed at restocking of exploited natural waters (Laikre, 1999). In Denmark, release of hatchery strain trout was a common management practice from the 1970s until the late 1990s where two partially domesticated and closely related hatchery strains were widely used to stock depleted rivers (Hansen et al., 2009). However, based on studies demonstrating substantial genetic structure among local wild populations coupled with a general expectation for hatchery strains to exhibit reduced fitness relative to indigenous fish (Hansen et al., 2002; Ruzzante et al., 2004), stocking with these hatchery strains was largely abandoned in favor of “supportive breeding” based on annually wild‐caught local brood stock. In general, the long‐term impacts of hatchery trout stocking is difficult to predict. Based on comparisons of genetic profiles in samples collected in the 1950'es (prior to stocking) and in the 1990'es (shortly after the termination of hatchery stocking), Hansen et al. (2002) showed that the genetic impact (i.e., the level of admixture in the native population) of stocking may vary significantly among populations. In some rivers, stocking resulted in almost complete displacement of the native population (strong introgression) whereas in others only small genetic changes were observed (weak introgression) even when massive numbers of fish had been stocked. Although local differences in the demographic state of the recipient population as well as stocking protocol may account for some of this variability (Hansen et al., 2009), it is possible that the observed variance in introgression reflects variance in the extent to which hatchery fish and their offspring display phenotypes conferring survival under wild conditions. Introgression rate is expected to vary with the stocked gene pool's degree of mal‐adaptation under wild conditions which conversely may vary with local environmental conditions (Baskett & Waples, 2013), for example to which the extent temperature regimes differ between wild and culture conditions and drive local selection pressures. Adaptation to temperature conditions is often implicated to be a driver of local adaptation and of population differentiation (Eliason et al., 2011), including in brown trout (Jensen et al., 2008). However, whether individuals exhibiting different levels of introgression also differ in their phenotypic response to temperature remains unknown for most salmonid species (Solberg et al., 2016), and in brown trout.

Here, we use an introgressed population of brown trout to investigate the effects of wild‐domesticated admixture (defined as the proportion of an individual's gene pool originating from the hatchery strain) on juvenile growth and survival under three temperature regimes. A common garden experimental design was established under standard hatchery conditions, partially allowing for the dissociation of heritable from environmentally induced phenotypic variation. Applying a factorial mating design and broodfish with three genetic backgrounds; hatchery strain, genetically pure wild fish, and naturally produced *n*th generation admixed fish, we test two hypotheses: first, if juvenile growth and survival differ between hatchery strain and genetically pure wild fish, with additive effects of admixture proportion on trait expression; and second, if growth, condition and survival reaction norms across temperature regimes show interaction with admixture classes.

## 2 MATERIALS AND METHODS

### 2.1 Stocking history of the River Varde

The River Varde (55°34′ N, 8°19′ E) flows into the eastern North Sea through the Wadden Sea and supports an anadromous brown trout population which has been exploited by net fisheries and anglers for more than 100 years. The first stocking was conducted in 1939 and was intensified from 1977 although the precise numbers of stocked fish were undocumented until 1987. In the period 1987–1997, a total of ~800,000 fish from two closely related hatchery strains were stocked into the river and its tributaries based on annual releases of juveniles and smolts (Hansen et al., 2009). The hatchery strains (hereafter collectively referred to as “HAT”) were founded mainly on broodstock originating from rivers draining into the Western Baltic Sea (Hansen et al., 1997), which constitutes the transitional waters between the brackish Baltic Sea and the fully saline North Sea. In spite of the strong environmental cline wild source populations from that area (separated from river Varde by approximately 700 km waterway) show low genome‐wide differentiation, with *F*_st_ estimated at 0.02–0.04 (Bekkevold et al., 2020). HAT was produced for the sole purpose of stock enhancement and was used to supplement wild trout populations throughout Denmark for several decades (Hansen et al., 1997, 2009). HAT was not subject to a formal selective breeding program; however, after at least five generations of closed captive propagation (1977–1997) we here assume the strain to be partly domesticated, following the definition of Hansen et al. (2002). Despite the survival of HAT fish in the wild being considerably lower than that of wild fish (Hansen et al., 2009 and references therein), stocking with HAT has resulted in introgression in several Danish populations. Since 1997, the River Varde has solely been subjected to supportive breeding using local wild caught brood fish (Peter Geertz‐Hansen, DTU Aqua, personal communication). Nearly two decades, translating into five generations (generation time 3.5 year; Hansen et al., 2002), after supplementation of the river Varde with HAT fish was terminated, the native population still displays considerable HAT introgression, with wild‐caught fish displaying various degrees of wild/HAT admixture (Bekkevold et al., 2024).

### 2.2 Brood stock

Forty‐six mature fish (TL 17–82 cm, average 49.6 cm) were caught by electrofishing in the River Varde on 25th Nov. 2012. In addition, six mature fish were obtained from the HAT strain previously used to stock the river. All fish were transferred to the *Danish Centre for Wild Salmon* hatchery in Skjern, where they were kept until ripening. Adipose fin tissue was sampled from brood fish, and their genetic background was determined as described in Bekkevold et al. (2024; also see Supplementary Material). Briefly, based on genotyping 3573 Single Nucleotide Polymorphisms, individual genome‐wide admixture proportion (*Q*) was assessed using the Bayesian clustering software STRUCTURE V2.3.4 (Pritchard et al., 2000) following Hansen et al. (2002). Theoretically, *Q*‐values ranged from 0, for pure non‐admixed wild fish, to 1, for pure HAT fish. Due to incomplete marker resolution, *Q* estimates varied within admixture classes, and brood fish were classified as respectively *wild* when *Q* < 0.2, *admixed* at *Q* 0.3–0.7, and HAT at *Q* > 0.8. Nine dams and seven sires were then selected, representing six *wild* (*Q* 0.02–0.19), five *admixed* (*Q* range 0.45–0.65) and five *HAT* (*Q* 0.97–0.99) brood stock classes. A sample of 10 unfertilized eggs was collected from dams, and egg wet and dry weights were measured to nearest *mg* (Table S2).

### 2.3 Experimental crosses

**FIGURE 1:**
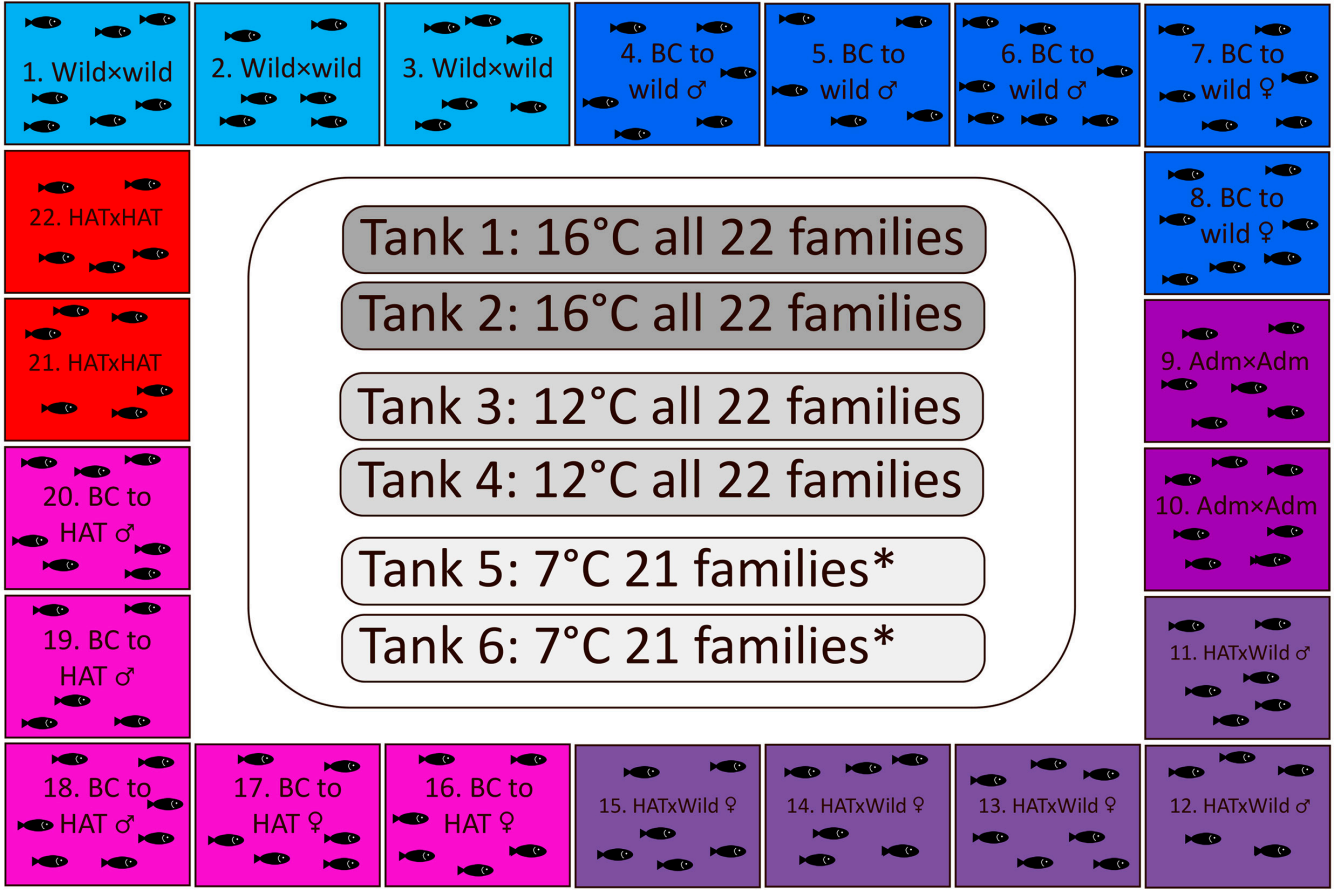
Common garden experimental setup. The 22 S. trutta families are shown by cross type and colour coded in increasingly red hues with increasing HAT admixture. Each of the six experimental tanks (with two replicates for each of the three temperature treatments) contained 34 fish from the 22 families (i.e. 748 fish per tank), except in the low temperature treatment (shown by *) that due to an error contained 21 families. Additional details on crosses are found in Table 1.

Brood fish were stripped and gametes were used to produce a mixture of 22 full‐sibling families, of which 13 were half‐sib families, using an hierarchical approach aimed at crossing each dam with multiple sires exhibiting different genetic backgrounds; wild, admixed and HAT. Due to the availability of ripe‐and‐running dams and sires, crosses were performed on three dates (18th Dec. 2012, 3rd and 10th Jan. 2013) (Table S3). Nine types of crosses with increasing levels of HAT admixture were generated: (i) “pure wild” (wild × wild), three families; (ii) “backcross to wild”‐sire (wild sire × admixed dam), three families; (iii) “backcross to wild”‐dam (wild dam × admixed sire), two families; (iv) “admixed” (admixed × admixed), two families; (v) “F1 hybrid”‐sire (wild sire × HAT dam), two families; (vi) “F1 hybrid”‐dam (wild dam × HAT sire), three families; (vii) “backcross to HAT”‐dam (admixed sire × HAT dam), two families; (viii) “backcross to HAT”‐sire (admixed dam × HAT sire), three families; (xi) “pure HAT” (HAT × HAT), two families (Figure 1; Table 1; Table S4). As a means to investigate the relationship between the investigated traits and genetic background, expressed as the degree of HAT admixture, sires and dams were assigned a proxy admixture coefficient of 0, 0.5 or 1 corresponding to wild, admixed and HAT genetic background. A family admixture coefficient calculated as the mean of sire and dam admixture was assigned to each family. Inference for cross types “F1 hybrid” and “admixed” were initially combined into one class exhibiting admixture coefficient 0.5.

**TABLE 1:** Summary statistics for individual brown trout families showing numbers of offspring analysed, *n*, and trait means for length (*L*), weight (*W*), condition factor (*K*), and survival (*S*) estimated as percentage individuals alive at sampling, across temperature treatments.

Family	Cross date	Dam ID	Sire ID	Family	Cross	7°C					12°C					16°C				
				Admixture	Type	*n*	*L* (cm)	*W* (g)	*K*	*S* (%)	*n*	*L* (cm)	*W* (g)	*K*	*S* (%)	*n*	*L* (cm)	*W* (g)	*K*	*S* (%)
1	18‐12‐2012	WD2	WS1	0	1	53	7.0	4.2	1.22	78	27	9.7	12.9	1.36	40	59	12.3	25.4	1.31	87
2	18‐12‐2012	WD2	WS2	0	1	60	7.0	4.0	1.18	88	66	10.3	14.9	1.27	97	56	13.5	32.9	1.29	82
3	03‐01‐2013	WD4	WS1	0	1	7	6.1	2.8	1.22	10	24	9.0	9.8	1.34	35	40	10.9	17.1	1.29	59
4	18‐12‐2012	AD5	WS2	0.25	2	33	6.9	4.0	1.18	49	45	11.8	22.8	1.33	66	35	15.5	51.1	1.35	51
5	03‐01‐2013	AD6	WS1	0.25	2	23	6.5	3.4	1.23	34	46	9.4	11.4	1.32	68	48	12.7	27.3	1.30	71
6	10‐01‐2013	AD7	WS2	0.25	2	21	5.7	2.1	1.10	31	46	8.5	7.9	1.24	68	39	12.9	27.5	1.24	57
7	10‐01‐2013	WD3	AS3	0.25	3	17	6.3	3.2	1.24	25	61	9.7	13.3	1.37	90	51	12.5	27.1	1.33	75
8	10‐01‐2013	WD1	AS3	0.25	3	29	5.8	2.4	1.26	43	50	9.3	11.7	1.37	74	59	12.5	25.7	1.30	87
9	18‐12‐2012	AD5	AS3	0.5	4	41	7.1	4.6	1.25	60	48	12.5	27.2	1.38	71	41	15.4	50.0	1.35	60
10	10‐01‐2013	AD7	AS3	0.5	4	26	5.7	2.2	1.20	38	45	9.7	12.2	1.28	66	34	12.8	27.1	1.25	50
11	18‐12‐2012	HD8	WS1	0.5	5	38	7.0	4.3	1.27	56	52	11.8	24.0	1.41	76	51	14.1	40.4	1.41	75
12	18‐12‐2012	HD8	WS2	0.5	5	44	7.2	4.5	1.20	65	59	12.9	29.3	1.32	87	59	15.2	48.6	1.36	87
13	18‐12‐2012	WD2	HS7	0.5	6	63	7.3	5.1	1.26	93	65	12.3	25.9	1.34	96	62	15.6	53.0	1.38	91
14	18‐12‐2012	WD2	HS5	0.5	6	63	7.7	5.6	1.23	93	66	13.3	30.9	1.32	97	60	14.7	42.7	1.31	88
15	10‐01‐2013	WD1	HS6	0.5	6	0^a^	NA	NA	NA	NA	13	9.5	11.8	1.32	19	30	13.0	31.0	1.35	44
16	18‐12‐2012	HD9	AS4	0.75	7	50	7.1	4.3	1.21	74	60	12.6	28.2	1.34	88	42	14.7	44.2	1.35	62
17	18‐12‐2012	HD9	AS3	0.75	7	47	7.2	4.7	1.26	69	61	13.3	33.0	1.35	90	54	14.7	45.9	1.39	79
18	18‐12‐2012	AD5	HS6	0.75	8	24	7.2	4.8	1.23	35	22	13.6	33.4	1.31	32	17	16.7	66.4	1.41	25
19	03‐01‐2013	AD6	HS5	0.75	8	38	6.6	3.8	1.26	56	64	12.8	28.0	1.31	94	51	14.3	38.7	1.31	75
20	10‐01‐2013	AD7	HS6	0.75	8	13	6.0	2.7	1.23	19	28	10.0	13.4	1.28	41	22	14.7	41.8	1.29	32
21	18‐12‐2012	HD8	HS7	1	9	35	6.6	3.8	1.33	51	51	13.7	37.0	1.42	75	56	17.0	77.4	1.50	82
22	18‐12‐2012	HD9	HS5	1	9	27	7.2	4.7	1.28	40	47	13.7	35.4	1.36	69	35	14.9	46.7	1.39	51

*Note*: Cross date, family admixture coefficient and cross type (used to denote crosses with increasing proportion HAT genetic input) is indicated, as well as Dam and Sire ID, where letters W (wild), A (admixed), and H (HAT) indicate genetic origin.

^a^ Due to error, family 15 was not included in the 7°C treatment and trait means are therefore denoted “NA”.

### 2.4 Ethics statement

The experimental protocol for rearing and sampling of fish was approved by the Norwegian Animal Research Authority (permit number 6447). All handling of experimental animals was done in accordance with the Norwegian Animal Welfare Act and the involved technical staff had been approved by the Norwegian Food Safety Authority. Permit for import of trout eggs was granted by the Norwegian Environment Agency (archive code 454.31/1) on January 9th 2013. Brood stock fish were released back into the River Varde, at the initial location of capture, after stripping of eggs and sperm.

### 2.5 Rearing conditions

Embryos were reared under standard hatchery conditions in separate incubators until the eyed egg stage. On 20th March 2013, eyed eggs were distributed into six replicate mixed family groups, where each of the 22 full‐sib families was represented by 34 embryos (thus 748 individuals per replicate). Due to an error, embryos from one family were not included in one of the six replicates (see below). Embryos were then transferred to the Norwegian Institute of Marine Research's salmonid experimental facility at Matre, Norway and incubated at 5°C until hatch and exogenous feeding. The common garden experiments were initiated on the 7th of May 2013. Temperature treatments were aimed at tracking natural, cold and warm conditions, respectively. Natural water temperatures in the River Varde show pronounced seasonal and annual variation varying 1.5–20.5°C during the first 8 months of juvenile phase, with an average for spring–summer months at ~12°C (Jepsen et al., 2003). 7°C was therefore chosen to test phenotypic response to cold conditions, as this temperature is within the range naturally experienced by the population but is close to the reported lower critical temperature for growth (Elliott & Hurley, 2000). 16°C was used to test response to warm conditions. Again, the chosen temperature is within the natural range of the wild population but is closer to average conditions for Danish strains in culture (Jokumsen & Svendsen, 2010). Replicated mixed‐family groups were thus subjected to temperature treatment at 7°C, 12°C or 16°C in 400 liter indoor tanks under standard rearing conditions, using a 24 h light regime and automated ad lib feeding with a standard salmonid feed. Dead fish were recorded and removed daily. In order to even out rearing densities across temperature treatments exhibiting highly varied growth rates, the two 16°C replicates were distributed into four tanks on 8th July 2013.

### 2.6 Sampling, genotyping, and parentage testing

The common garden experiment was terminated after 23 weeks of rearing, on 14th October 2013. All fish (*N* = 2799) were euthanized with an overdose of metacain (Finquel Vet, ScanVacc, Årnes, Norway). Fork length (*L*) and wet weight (*W*) were measured and fin tissue was sampled and preserved in 95% EtOH. DNA was extracted from 2799 offspring and 16 brood stock fish in 1.5 mL Eppendorph tubes using a 10% Chelex® 100 resin solution (BIO‐RAD) following the protocol recommended by the manufacturer. DNA was amplified in one multiplex PCR (Qiagen) for seven microsatellite loci: Ssa85, Ssa197 (O'Reilly et al., 1996), SsOsl417 (Slettan et al., 1995), Ssa24NVH, Ssa87NVH (Gharbi et al., 2006), BS131 (Estoup et al., 1998), Ssa408 (Cairney et al., 2000). Genotyping was done on the ABI Applied Biosystems ABI 3130 Genetic Analyzer using 500LIZ size standards (GeneScan™) followed by manually verified scoring of alleles in the software GeneMapper V4.0. The software COLONY V2.0.5.0 (Jones & Wang, 2010) was used to assign parentage. Fifteen individuals could not be unambiguously assigned to family based on the seven markers, and were therefore genotyped for three additional microsatellite markers (Ssa54NVH, Ssa94NVH, and Ssa64NVH from Gharbi et al. (2006)). In four individuals, one or several loci exhibited three alleles. These fish were also analyzed with the additional markers and their parentage was manually deduced from brood fish genotypes. All four trisomic individuals shared the same hatchery mother. As their lengths and weights fell within the 2nd and 3rd quartiles of the *L* and *W* distributions for that particular female half‐sib family and respective temperatures, they were considered valid and included in the dataset.

### 2.7 Statistical analyses

#### 2.7.1 Length, weight, and the condition factor

The condition factor (*K*) was calculated from individual length (*L*) and weight (*W*) according to Fulton's formula (Ricker, 1975),

$$K=100^{*}\left(W/L^{3}\right).$$

The effect of genetic background on *L*, log(*W*) and *K* was examined using linear mixed effects models (LME) implemented in the “lmer” function in R (Bates et al., 2015). Akaike information criterion (AIC) were used in model selection.

During the model selection process, models for response variables *L*, log(*W*) and *K* were tested, including fixed effects of admixture (*A*), temperature treatment (*T*), fertilization date (*D*), and replicate (*R*). The effect of fertilization date (*D*) was included to account for the effect of three different fertilization dates on the final body size, however due to the overlap between admixture and date (certain cross types only occurred on 1 day), the interactions between date and other cofactors were not explored. In addition, the random effects of Sire, Dam, and Tank were considered. Based on the AIC (Tables S6–S9), the following model was retained for length:

$$\text{Length}=\beta \left(A^{*}\;T+D\right)+\text{Sire}+\mathrm{Dam}+\text{Tank}+\varepsilon$$

where (A*T + *D*) is the model matrix for the marginal fixed effects of admixture, temperature and fertilization date as well as the interaction term between admixture and temperature. Sire, Dam, Tank, and *ε*, respectively, denote the random effects of the parents, tank, and random residuals Sire ~ *N*(0,σ^2^), Dam ~ *N*(0,σ^2^) and ε ~ *N*(0,σ^2^), all independent.

Similarily, the retained model for log(*W*) was:

$$\mathrm{Log}\left(W\right)=\beta \left(A\times T+D\right)+\text{Sire}+\mathrm{Dam}+\text{Tank}+\varepsilon$$

and for *K*:

$$K=\beta \left(A^{*}\;T+D\right)+\varepsilon$$

#### 2.7.2 Survival

Differences in survival were examined using a logit‐linear binomial model with random effects (GLMM) implemented in the “glmer” function. The respective proportions (*p*) of live fish in each family at experiment termination were entered into the model as response variable, and model selection was applied with the same covariates as for the growth model (Table S8). The selected model for survival was thus:

$$\text{logit}\left(p\right)=\beta \left(T+D\right)+\text{Sire}+\mathrm{Dam}+\text{Tank}+\varepsilon$$

where (*T* + *D*) is the model matrix for the fixed effects of temperature and fertilization date. Sire, Dam, Tanks and *ε*, respectively, denote the random effects of the parents, tank and random residuals Sire ~ *N*(0,σ^2^), Dam ~ *N*(0,*σ* ^2^) and *ε* ~ *N*(0,*σ* ^2^), all independent.

## 3 RESULTS

### 3.1 Experimental family data

Across all replicates and treatments, phenotypic data were available for an average of 127 offspring per family. In general, survival was high in the experiment but significantly lower at the low temperature treatment (*df* = 2, *χ* ^2^ = 33, *p* < 0.01, Table S5; Figure S1), leading to variability in the total number of fish analyzed per treatment (752, 1046 and 1001 for 7, 12 and 16°C, respectively). In all models, fertilization date had a significant effect on size at sampling, reflecting that earlier crosses had a longer growth period (Table 1).

### 3.2 Growth

**FIGURE 2:**
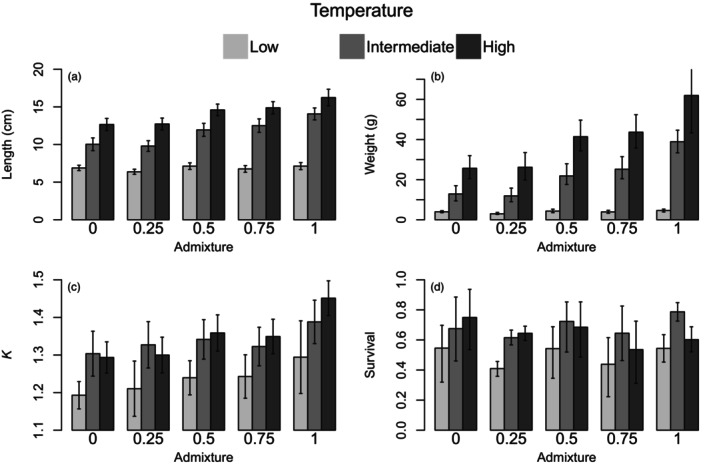
Model predictions of length (a), weight (b) condition factor (Fulton's *K*) (c), and survival (d) for each class of temperature treatment, and admixture. The variability around the point estimates is represented as the standard deviation of the residuals.

As expected, growth was strongly linked with temperature. This trend was statistically significant in both pairwise comparisons between intermediate and low temperature treatment (*df* = 1, *χ* ^2^ = 25, *p* < 0.01), and between high and intermediate treatment (*df* = 1, *χ* ^2^ = 24, *p* < 0.01, Figure 2). On average, fish in the low temperature were 4.1 ± 1.3 g upon termination of the experiment whereas fish in the high temperature treatment were on average 40.0 ± 18 g.

A statistically significant positive correlation was observed between growth, as measured by size upon termination, and family admixture (*df* = 1, χ^2^ = 20, *p* < 0.01, Tables S6 and S7), as highly admixed families grew on average 36 ± 9 g versus 13 ± 7 g for the low admixed families in the intermediate (12°C) treatment. The effect of admixture was not equal among temperature treatments, as illustrated by the significant admixture*treatment interaction term of the growth model (*df* = 4, *χ* ^2^ = 17, *p* < 0.01). This interaction can be observed in Figure 2, where size as measured by *length* (Figure 2a) and *weight* (Figure 2b) increases with admixture in the high and intermediate temperature treatment (*df* = 1, *χ* ^2^ = 734, *p* < 0.01), whereas the correlation between admixture and growth is weaker, however still statistically significant, in the low temperature treatment (*df* = 1, χ^2^ = 7.9, *p* < 0.01).

Similar to *length* and *weight*, condition factor *K* revealed a correlation with temperature and admixture type (Figure 2c), with a statistically significant increase in *K* with temperature (*df* = 2, *χ* ^2^ = 23, *p* < 0.01, Table S8) and admixture (*df* = 1, *χ* ^2^ = 173, *p* < 0.01). The effect of temperature was mainly caused by low *K* values in the low temperature treatment however, and no difference in *K* was found between the intermediate and high temperature treatments (*df* = 1, *χ* ^2^ = 1.2, *p* = 0.26). *K* estimates were independent of individual parents' contribution; therefore, the random effect of sire and dam was not included in the model for *K*.

### 3.3 Survival

Survival was correlated with temperature and fertilization date with lower survival observed among the low temperature treatment (*df* = 2, *χ* ^2^ = 33, *p* < 0.01, Figure 2d; Table S9). The difference in survival between treatments was due to a higher mortality rate in the low temperature treatment group, but the difference in survival was non‐significant between intermediate and high temperature treatment groups (*df* = 1, *χ* ^2^ = 0.5, *p* = 0.46). No correlation between admixture and survival (*df* = 1, *χ* ^2^ = 0.43, *p* = 0.51) was observed.

## 4 DISCUSSION

Understanding population level fitness consequences of admixture with non‐local, hatchery and/or domesticated organisms is important in a world where anthropogenic factors are increasingly challenging natural resources. With the exception of studies in Atlantic salmon, where decades of extensive aquaculture escapes have left a legacy of interaction with wild populations (Glover et al., 2017), data on the fitness consequences of admixture from non‐local conspecifics are still quite rare. In order to address this, we used a common‐garden experimental design to study growth and survival of brown trout offspring originating from wild captured individuals that exhibit different levels of admixture with a partially domesticated hatchery strain that was used to stock the population two decades prior to the study. The three main results of our study can be summarized as follows: (i) The hatchery strain historically used to stock multiple wild Danish trout populations displayed increased growth relative to the wild population, and there was an additive effect of HAT admixture on growth. This is concordant with observations in domesticated/wild admixed populations of other salmonid species. Our results therefore demonstrate that genetic changes in phenotypic traits, such as growth, can occur in wild populations subjected to admixture with hatchery fish, even when the strain in question has not been subjected to directional selection. (ii) HAT admixture affected juvenile phenotypes close to two decades after cessation of direct release of HAT fish into the population. During this time, the admixed component of the population had been subject to natural selection, but genetic capacity for higher growth had not been purged to the extent that it is not detectable several generations later. (iii) The identification of a significant interaction with temperature shows that phenotypic response to temperature differed among fish exhibiting different levels of admixture, and suggests that all other factors being equal, admixed fish may show increased phenotypic divergence from wild‐type growth patterns under increasing temperature regimes in the wild.

### 4.1 Effect of temperature and admixture on growth

Fish metabolism and developmental rates are sensitive to temperature, and temperature conditions experienced during juvenile stages can under certain conditions be detected in the phenotype throughout an individual's lifetime (Auer et al., 2012). Temperature adaptation has been identified as one of the potential drivers of local adaptation in salmonids (Garcia de Leaniz et al., 2007), also among brown trout populations (Jensen et al., 2008 but see Bærum et al., 2016). Our experimental design allowed the assessment of growth‐related responses to temperature regimes ranging from close to the lower critical temperature for growth (Elliott & Hurley, 2000; Ojanguren et al., 2001; Rogell et al., 2012) to relatively warm conditions expected to be closer to those often experienced in culture (Jokumsen & Svendsen, 2010). Our results demonstrated a clear negative effect of low temperature on growth and survival, and maximal growth under high temperature, irrespective of admixture, as also demonstrated in Atlantic salmon of wild, hybrid and domesticated origin (Harvey et al., 2016; Solberg et al., 2016).

Growth rate increases rapidly under domestication when combined with directional selection for this trait, as has been well documented in Atlantic salmon (reviewed in Glover et al., 2017). The hatchery strain investigated in the present study had been cultured for at least seven generations. During this time there was no deliberate directional growth or size selection in place, although hatchery procedures for choosing brood fish likely entailed some undisclosed preference for larger, potentially fast‐growing fish as seen in other species (e.g. LaCava et al., 2023). Notwithstanding, our study demonstrated an increase in growth, as measured by size, associated with increasing HAT admixture, thus demonstrating a genetically increased growth rate. Whether the enhanced growth rate in HAT is an effect resulting from inadvertent selection in the hatchery or an attribute of the genetic makeup of the brood stock initially used to establish HAT could not be determined here. It nevertheless contributes to literature demonstrating that hatchery supplementation, also without clear directional selection regimes, causes heritable phenotypic changes (Araki et al., 2007, 2008). The fact that individual admixture level was inferred from estimates in broodstock means that our estimates of admixture‐growth relationships are conservative, giving even more weight to the argument of clear functional significance of introgression through its affect on phenotypic traits.

A large body of literature documents genetic changes in salmonid populations reared in culture, subjected to inadvertent domestication, or to domestication combined with directional selection (Reviewed in Glover et al., 2017). However, with the exception of recent studies in Atlantic salmon (Besnier et al., 2022; Bolstad et al., 2017, 2021), direct evidence of heritable changes in phenotypic traits of wild populations subjected to admixture with domesticated conspecifics are still sparse. In this regard, the main novelty of our study is that we investigated growth in the offspring of naturally produced brown trout displaying admixture with hatchery‐strains, resulting from releases that were terminated 15 years previously. Thus, depending on their individual life histories and hybridisation histories, the admixed broodstock used here had undergone no less than four generations of propagation in the wild, retaining genome‐wide admixed genetic profiles. However, although these fish had been exposed to 4–5 generations of natural selection, which is likely to purge strongly mal‐adapted phenotypes from the wild (Castellani et al., 2018; McGinnity et al., 2003), the genetically improved growth capacity was still detectable.

An aspect of our experimental set‐up was that crosses differed in age by up to 23 days. This resulted in variance in growth periods among maternal families, and as a result, older fish displayed larger size and hence potentially had a competitive advantage and improved survival that could have impacted trait expression beyond mere age differences (see Figures S2 and S3). However, all nine cross types were represented among families fertilized on the first date, which dampened potential age effects in the models used. Furthermore, and significantly, *L* and *W* estimates within maternal half‐sib crosses (i.e. crosses fertilized on the same date) consistently showed a positive relationship with family admixture. We therefore do not expect age differences to have significantly biased inference of a relationship between admixture and growth parameters. The fact that fertilization date was not significant in the condition model further suggests that not all traits were affected by age variation.

### 4.2 Effect of temperature and admixture on survival

In salmonids, size is often positively correlated with survival, particularly during early life stages (Debes & Hutchings, 2014; Sogard, 1997). Our results showed improved survival in the 12 and 16°C treatments compared with the 7°C treatment, and daily mortality rates generally decreased with increasing temperature (Figure s1). However, despite survival rates greatly varying among families, we observed no overall effect of admixture on survival probability, in consistency with other studies conducted under hatchery conditions (e.g. Debes et al., 2013; Solberg et al., 2016). The lack of relationship between family growth and family survival in the present study may reflect relatively low competition under experimental conditions (Rogell et al., 2012) and the overall high survival observed here. Studies in the natural habitat however, clearly demonstrate reduced survival of domesticated or admixed fish in comarison with wild conspecifics (Fleming et al., 2000; McGinnity et al., 2003; Skaala et al., 2012). Importantly, the lack of association observed between cross type and survival implies that our estimates of growth related traits were unlikely to suffer strong bias potentially induced by non‐random survival.

### 4.3 Predictions for responses under wild conditions

The observation that there was an additive effect of admixture on juvenile growth fuels the concern that stocked HAT fish and their descendants may have a competitive advantage over their wild counterparts under some environmental conditions. However, relationships between stage‐specific growth rates, growth potential and life‐time fitness in trout remain unclear (Bærum et al., 2016) and juvenile growth rates need not reflect lifetime fitness (Dmitriew, 2011; McGinnity et al., 2003). Domestication and large growth potential has been connected with greater risk taking behavior and susceptibility to predation in salmon (Solberg et al., 2020), and the lower growth rates observed in genetically pure wild crosses may thus reflect trade‐offs with other fitness traits (Biro et al., 2004, 2006; Debes & Hutchings, 2014; Rogell et al., 2012; Sundström et al., 2005; Tomchyk et al., 2006). Trout populations exhibit large variation in life‐history traits including migratory and maturation strategies and timing (reviewed in Jonsson & Jonsson, 2011); traits which in Atlantic salmon have been reported to show strong heritability (Ayllón et al., 2015; Barson et al., 2015; Lepais et al., 2017) and co‐variance with juvenile growth rates (Piché et al., 2008). If such traits are under selective control under wild conditions, this presents a possible causal link between introgression‐induced growth changes and observed fitness reductions in wild populations (see also Bolstad et al., 2017). Conditions for, and speed of, purging of mal‐adaptive traits in introgressed populations are expected to depend on a suite of parameters (e.g. Castellani et al., 2018). Our study did not allow for assessment of whether juvenile growth parameters are under selection, but merely point to the fact that admixed populations exhibit heritable variance that may be mal‐adaptive in the wild. Genetic monitoring of the Varde population supports the notion that natural selection still acts against hatchery genes seven generations after stocking ended (Bekkevold et al., 2024).

Climate‐change mediated increases in water temperature are predicted to increase local extinction rates of salmonids (Almodóvar et al., 2012; Jonsson & Jonsson, 2009), and introgressed populations may show different responses to climate change than non‐introgressed populations (Debes et al., 2021; Muhlfeld et al., 2014). Our findings support that responses to increasing temperature are likely to differ among individuals exhibiting different levels of introgression. If increased juvenile growth in fact incurs a competitive advantage at one or more life stages, climate change may potentially influence introgression effects in natural populations. Our study was performed under hatchery conditions, begging the question as to whether these results are predictive of mechanisms under wild conditions. Evidence from comparative studies shows that although reaction norms and heritability of traits examined in culture may not be directly applicable for predictions under natural dynamics (e.g. Vasemägi et al., 2016), genotype‐by‐environment interaction need not always impair inference from experimental studies (Rogell et al., 2012). Our findings emphasize that introgression by domesticated gene pools can significantly alter important biological characteristics of wild populations.

## CONFLICT OF INTEREST STATEMENT

Authors declare no conflicts of interest.

## Supporting information


Data S1

## Data Availability

Data underlying this article are available as online Supplementary Material (containing Tables S1–S9 and Figures S1–S3) and phenotype data for all fish are found in the DTU Data repository at https://data.dtu.dk/00000 with the DOI (to be completed after manuscript is accepted for publication).
