# Supplementary material for: Introgression affects Salmo trutta juvenile life‐history traits generations after stocking with non‐native strains
Source: Evol Appl. 2024 Jul 2;17(7):e13725. doi: 10.1111/eva.13725 (PMC11219512; doi:10.1111/eva.13725)
Supplement: Supplementary file 1 — Data S1 [file EVA-17-e13725-s001.docx]

# Supplementary material to “*INTROGRESSION AFFECTS SALMO TRUTTA JUVENILE LIFE-HISTORY TRAITS GENERATIONS AFTER STOCKING WITH NON-NATIVE STRAINS*”

**Admixture classification in broodstock fish**

Genetic marker data were available for all broodstock fish used in crosses from a population study reported in Bekkevold et al. (2020). Briefly, Bekkevold et al. used a 3.8K candidate Illumina iSelect SNP array to describe genome wide genetic variation in trout populations across the Northeast Atlantic. SNPs represented all 40 trout LGs with an average of 95 SNPs per LG (range 13–173). Individual broodstock fish HAT/wild admixture was determined in an admixture analysis with program *structure* v.2.3.4 (Pritchard et al. 2000) using genotypes for all available samples of adult spawners collected from River Varde during the spawning season 2012 (N=44) and previously collected HAT fish (N=74). *structure* trial runs for 2-5 K clusters showed best classification into two clusters corresponding to HAT and wild genotypes. In analyses allele frequencies were assumed correlated, and five replicate runs (to check consistency) were performed using 100K MCMC (discarding the first 50K MCMC as burn-in). Pre-stocking samples of fish of non-admixed pure wild origin were not available. Individuals were therefore initially classified as pure wild or admixed based on their *q* estimate and 90% CI based from *structure* analyses of 3573 SNPs segregating in the subset of samples from Bekkevold et al. (2020). Individuals with Q < 0. 2 were classified as pure wild genotypes. Analyses showed that all samples obtained directly from the hatchery (HAT) always exhibited lower 90% CI for Q > 0.80. Individuals with *q* ranging 0.20-0.80 were therefore initially classified as being of admixed origin. The validity of this classification and the potential to classify individuals displaying different levels of admixture were tested by simulation. Using allele frequency estimates for 74 HAT and for 24 wild caught fish with Q < 0.20, multi-locus genotypes were simulated using the function ‘hybridize’ in *adegenet* as to correspond with five admixture classes with increasing HAT admixture: 1) Wild, 2) Back-crossed to wild, BC_WILD_ (F1 x WILD cross), 3) F1 (WILD x HAT cross), 4) Back-crossed to HAT, BC_HAT_ (F1 x HAT cross), and 5) HAT. A total of 50 genotypes were simulated per admixture class, resulting in 250 in total. Simulated genotypes were examined in an admixture analysis using the same settings as above, and range and mean q values together with 90% CI were compared among classes. Finally, based on specific Q thresholds observed in simulated data, all wild-caught fish were classified as either ‘Wild’, ‘low admixture’ (n^th^ generation back-crossed to wild), ‘medium admixture’ (n^th^ generation admixed cross), ‘high admixture’ (n^th^ generation back-crossed to HAT), or ‘HAT’. In simulations, admixture samples showed Q values close to theoretical expectations for each admixture class and Q estimates did not overlap between admixture classes, whereas 90% CI sometimes overlapped. From this analysis broodstock fish were classified as shown in Table S1. Further details of the analyses are available in Bekkevold et al. (under submission).

Table S1. Overview of admixture data for simulated and observed genotypes in river Varde, showing criteria for classifications of wild-caught broodstock fish as wild, admixed and HAT.

| Simulated admixture class | Expected mean Q | average Q [90% CI range] | Classification criteria for broodstock fish shown as Q range | Q estimates for broodstock fish (by individual ID) |
| --- | --- | --- | --- | --- |
| Non-admixed pure wild | 0 | 0.02 [0.00-0.12] | 0.00-0.12 | 0.05 (WS1), 0.12 (WS2), 0.02 (WD1), 0.09 (WD2), 0.05 (WD3), 0.03 (WD4), |
| Admixed: BC_WILD_ | 0.25 | 0.21 [0.07-0.34] | 0.13-0.34 | - |
| Admixed: F1 | 0.50 | 0.47 [0.35-0.60] | 0.35-0.60 | 0.49 (AS4), 0.45 (AD5) |
| Admixed: BC_HAT_ | 0.75 | 0.74 [0.61-0.85] | 0.61-0.84 | 0.62 (AS3), 0.64 (AD6), 0.63 (AD7) |
| HAT | 1 | 0.98 [0.84-1.00] | 0.84-1.00 | 0.99 (HS5), 0.96 (HS6), 0.99 (HS7), 0.99 (HD8), 0.97 (HD9) |

**Literature cited here:**

Bekkevold, D., K.A. Glover, the Aquatrace Consortium, B. Jimenez-Mena, E.E. Nielsen (.) Signatures of genetic recovery from hatchery stocking in *Salmo trutta* trout (manuscript under submission).

Bekkevold, D., Hojesjo J., Nielsen E.E., et al. (2020) Northern European Salmo trutta (L.) populations are genetically divergent across geographical regions and environmental gradients. Evolutionary Applications. 13: 400-416.

Pritchard, J. K., Stephens, M., and Donnelly, P. (2000) Inference of population structure using multilocus genotype data. Genetics, 155: 945–959.

Table S2. Mean egg dry weight (mg) for each dam (D), where letters W (wild), A (admixed), and H (HAT) indicate genetic origin.

| **Dam ID** | **Egg dry weight** |
| --- | --- |
|  | **(mg)** |
| WD1 | 29.2 |
| WD2 | 31.8 |
| WD3 | 25.2 |
| WD4 | 30.8 |
| AD5 | 24.6 |
| AD6 | 24.3 |
| AD7 | 19.2 |
| HD8 | 22.2 |
| HD9 | 20.1 |

Table S3. Cross matrix showing family crosses produced from the 16 brood fish (9 dams and 7 sires). W (wild), A (admixed) and H (HAT) indicate genetic background and D (dam) and S (sire) indicate sex. Percent HAT admixture and 90% confidence intervals estimated with *structure* is indicated in brackets for each brood fish. Numbers in square bracets show total length in cm.

| **Sire/dam ID** | WD1 | WD2 | WD3 | WD4 | AD5 | AD6 | AD7 | HD8 | HD9 |
| --- | --- | --- | --- | --- | --- | --- | --- | --- | --- |
|  | (2; 0-6)  [58] | (9; 0-17)  [55] | (5; 0-14)  [48] | (3; 0-10)  [58] | (45; 36-53)  [51] | (64; 56-71)  [48] | (63; 55-71)  [49] | (99; 98-100)  [47] | (97; 91-100)  [44] |
| WS1 (5; 0-11) [71] |  | X |  | X |  | X |  | X |  |
| WS2 (19; 8-28) [48] |  | X |  |  | X |  | X | X |  |
| AS3 (62; 55-69) [50] | X |  | X |  | X |  | X |  | X |
| AS4 (49; 42-55) [49] |  |  |  |  |  |  |  |  | X |
| HS5 (99; 96-100) [48] |  | X |  |  |  | X |  |  | X |
| HS6 (96; 90-100) [48] | X |  |  |  | X |  | X |  |  |
| HS7 (99; 98-100) [49] |  | X |  |  |  |  |  | X |  |

Table S4. Cross types and family admixture categories of the 22 full sib families indicating the number of families and reciprocal crosses in each category.

| **Cross type** | **Family admixture** | **No. of families** |
| --- | --- | --- |
| Pure wild (wild x wild) | 0 | 3 |
| Backcross to wild (admixed ♂ x wild ♀) | 0.25 | 2 |
| Backcross to wild (admixed ♀ x wild ♂) | 0.25 | 3 |
| Admixed (admixed x admixed) | 0.5 | 2 |
| Hybrid (wild ♂ x HAT ♀) | 0.5 | 2 |
| Hybrid (wild ♀ x HAT ♂) | 0.5 | 3 |
| Backcross to HAT (admixed ♂ x HAT ♀) | 0.75 | 2 |
| Backcross to HAT (admixed ♀ x HAT ♂) | 0.75 | 3 |
| Pure HAT (HAT x HAT) | 1 | 2 |

Table S5. Summary statistics of length (*L*), weight (*W*), condition factor (*K*) and survival (*S*) as well as number of sampled juveniles (*n*) summed over all family crosses, shown for each temperature treatment and replicate tank.

|  |  |  |  |  |  |  |  |  |  |  |  |  |
| --- | --- | --- | --- | --- | --- | --- | --- | --- | --- | --- | --- | --- |
|  |  |  | ***L* (cm)** | |  | ***W* (g)** | |  | ***K*** | |  |  |
| **Treatment** | **Replicate** | ***n*** | **mean** | **sd** |  | **mean** | **sd** |  | **mean** | **sd** |  | ***S* (%)** |
| 7 °C | 1 | 383 | 6.9 | 0.8 |  | 4.2 | 1.4 |  | 1.25 | 0.10 |  | 51 |
|  | 2 | 369 | 6.9 | 0.7 |  | 4.2 | 1.3 |  | 1.21 | 0.07 |  | 49 |
| 12 °C | 1 | 539 | 11.4 | 2.1 |  | 21.4 | 10.9 |  | 1.32 | 0.09 |  | 72 |
|  | 2 | 507 | 11.7 | 2.2 |  | 24.0 | 12.2 |  | 1.35 | 0.09 |  | 68 |
| 16 °C | 1 | 476 | 14.3 | 2.0 |  | 42.0 | 19.0 |  | 1.35 | 0.09 |  | 64 |
|  | 2 | 514 | 13.9 | 1.9 |  | 38.1 | 17.9 |  | 1.33 | 0.09 |  | 69 |

**Table S6**: a) AIC values for fixed and random parts of the growth model (using length as response variable). Admixture and temperature are treated as a numerical variables, cross-date and replicate as factorial variables. AIC for the random effects are computed from models accounting for retained fixed effects. b) The significance of each model covariate in Type III Analysis of Variance Table with Satterthwaite's method, using lengt as response variable.

| a) Effects type | Effects list | AIC |
| --- | --- | --- |
| Fixed effects | 1 | 14635 |
|  | 1+Admixture | 14470 |
|  | 1+Temperature | 11126 |
|  | 1+Cross-date | 14552 |
|  | 1+replicate | 14636 |
|  | 1+Admixture+temperature+cross-date | 9548 |
|  | 1+Admixture* temperature +cross-date | 9213* |
| Random effects | Tank | 9087 |
|  | Dam | 9005 |
|  | Sire | 9001 |
|  | Sire+dam | 8954** |

*retained fixed effects, ** retained random effects

| b) Effects list | Sum Sq | Mean Sq | DF | DenDF | F value | Pr(>F) |
| --- | --- | --- | --- | --- | --- | --- |
| Admixure | 41.19597 | 41.19597 | 1 | 9.877093 | 29.24849 | 0.000311 |
| Cross_date | 33.23991 | 16.61996 | 2 | 8.502963 | 11.79991 | 0.003524 |
| Temperature | 1859.489 | 929.7446 | 2 | 2762.89 | 660.104 | <0.00001 |
| Admix:cross_date | 1.973522 | 0.986761 | 2 | 807.6619 | 0.700585 | 0.496596 |
| Admix:Temp | 165.4096 | 82.7048 | 2 | 2762.807 | 58.7191 | <0.00001 |
| Cross_date:Temp | 22.27236 | 5.568091 | 4 | 2762.205 | 3.953256 | 0.003338 |
| Admix:Cross_date:Temp | 31.11521 | 7.778802 | 4 | 2762.963 | 5.522827 | 0.000199 |

**Table S7**: a) AIC values for fixed and random parts of the growht model (using log(weight) as response variable). AIC for the random effects are computed from models accounting for retained fixed effects. b) The significance of each model covariate in Type III Analysis of Variance Table with Satterthwaite's method, using log(weight) as response variable.

| a) Effects type | Effects list | AIC |
| --- | --- | --- |
| Fixed effects | 1 | 7967 |
|  | 1+Admixture | 7835 |
|  | 1+Temperature | 3926 |
|  | 1+Cross-date | 7899 |
|  | 1+Replicate | 7969 |
|  | 1+Admixture+temperature+cross-date | 2236 |
|  | 1+Admixture*temperature+cross-date | 1992* |
| Random effects | Tank | 1921 |
|  | Dam | 1823 |
|  | sire | 1863 |
|  | Sire+dam | 1801** |

*retained fixed effects, ** retained random effects

|  |  |  |  |  |  |  |
| --- | --- | --- | --- | --- | --- | --- |
|  |  |  |  |  |  |  |
| \| b) Effects list \| Sum Sq \| Mean Sq \| DF \| DenDF \| F value \| Pr(>F) \| \| --- \| --- \| --- \| --- \| --- \| --- \| --- \| \| Admixure \| 5.392233 \| 5.392233 \| 1 \| 7.263067 \| 50.9382 \| 0.000157 \| \| Cross_date \| 3.146987 \| 1.573494 \| 2 \| 8.21706 \| 14.8641 \| 0.001863 \| \| Temperature \| 144.9401 \| 72.47006 \| 2 \| 19.76345 \| 684.594 \| <0.00001 \| \| Admix:cross_date \| 0.01495 \| 0.007475 \| 2 \| 602.1261 \| 0.07061 \| 0.931832 \| \| Admix:Temp \| 9.085692 \| 4.542846 \| 2 \| 2760.413 \| 42.9143 \| <0.00001 \| \| Cross_date:Temp \| 1.018352 \| 0.254588 \| 4 \| 2758.59 \| 2.40498 \| 0.0476 \| \| Admix:Cross_date:Temp \| 1.805176 \| 0.451294 \| 4 \| 2759.804 \| 4.26318 \| 0.001927 \| |  |  |  |  |  |  |
|  |  |  |  |  |  |  |
|  |  |  |  |  |  |  |
|  |  |  |  |  |  |  |
|  |  |  |  |  |  |  |
|  |  |  |  |  |  |  |

**Table S8**: a) AIC values for fixed and random parts of the growht model (using *K* as response variable). AIC for the random effects are computed from models accounting for retained fixed effects. b) The significance of each model covariate in Type III Analysis of Variance Table with Satterthwaite's method, using *K* as response variable.

| a) Effects type | Effect list | AIC |
| --- | --- | --- |
| Fixed effects | 1 | -1361 |
|  | 1+Admixture | -1421 |
|  | 1+Temperature | -1497 |
|  | 1+Cross-date | -1373 |
|  | 1+Replicate | -1359 |
|  | 1+Admixture+temperature+cross-date | -1578 |
|  | 1+Admixture*temperature+cross-date | -1584* |
| Random effects | Tank | -1486 |
|  | Dam | -1523 |
|  | sire | -1538 |
|  | Sire+dam | -1545 |

*retained fixed effects

| b) Effects list | Sum Sq | Mean Sq | DF | DenDF | F value | Pr(>F) |
| --- | --- | --- | --- | --- | --- | --- |
| Admixure | 0.02463 | 0.02463 | 1 | 9.965389 | 3.925272 | 0.075817 |
| Cross_date | 0.017003 | 0.008502 | 2 | 8.010113 | 1.35489 | 0.311281 |
| Temperature | 0.915591 | 0.457795 | 2 | 2761.621 | 72.9575 | <0.00001 |
| Admix:cross_date | 0.022888 | 0.011444 | 2 | 1692.865 | 1.823802 | 0.161728 |
| Admix:Temp | 0.150827 | 0.075414 | 2 | 2761.093 | 12.01843 | <0.00001 |
| Cross_date:Temp | 0.056066 | 0.014016 | 4 | 2761.381 | 2.233754 | 0.063032 |
| Admix:Cross_date:Temp | 0.067267 | 0.016817 | 4 | 2761.472 | 2.680028 | 0.03011 |

**Table S9**: a) AIC values for fixed and random parts of the survival model. AIC for the random effects are computed from models accounting for retained fixed effects. b) The significance of each model covariate in Analysis of Deviance Table (Type II Wald chisquare tests) using survival as variable.

| a) Effect type | Effect list | AIC |
| --- | --- | --- |
| Fixed effects | 1 | 6204 |
|  | 1+Admixture | 6205 |
|  | 1+Temperature | 6078 |
|  | 1+Replicate | 6206 |
|  | 1+Cross-date | 6021 |
|  | 1+Cross-date+Temperature | 5890* |
| Random effects | Tank | 5471 |
|  | Dam | 5588 |
|  | Sire | 5563 |
|  | Tank+dam | 5215 |
|  | Tank+sire | 5204 |
|  | Tank+dam+sire | 5030** |

*retained fixed effects, ** retained random effects

| b) Effect | Chisq | DF | Pr(>Chisq) |
| --- | --- | --- | --- |
| Admixture | 0.531197 | 1 | 0.466104 |
| Temperature | 146.2543 | 2 | <0.00001 |
| Cross_date | 6.369448 | 2 | 0.04139 |
| Admixture:Temperature | 24.73438 | 2 | <0.00001 |


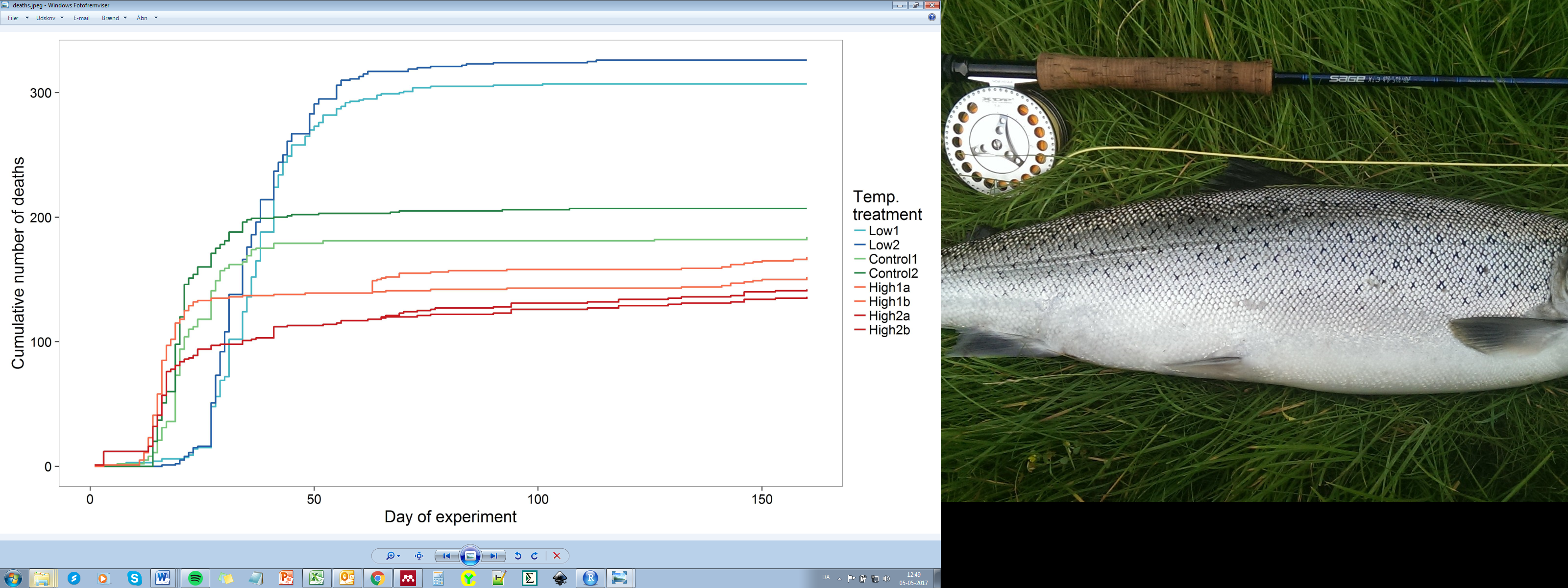


Figure S1. Cumulative number of deaths for each of the three temperature treatments (Low = 7 °C, Control = 12 °C, High = 16 °C) and two replicates per treatment. Note that to avoid crowding, each of the two replicates at 16 °C were further subdivided into two tanks on day 63 of the experiment (red and light red lines).


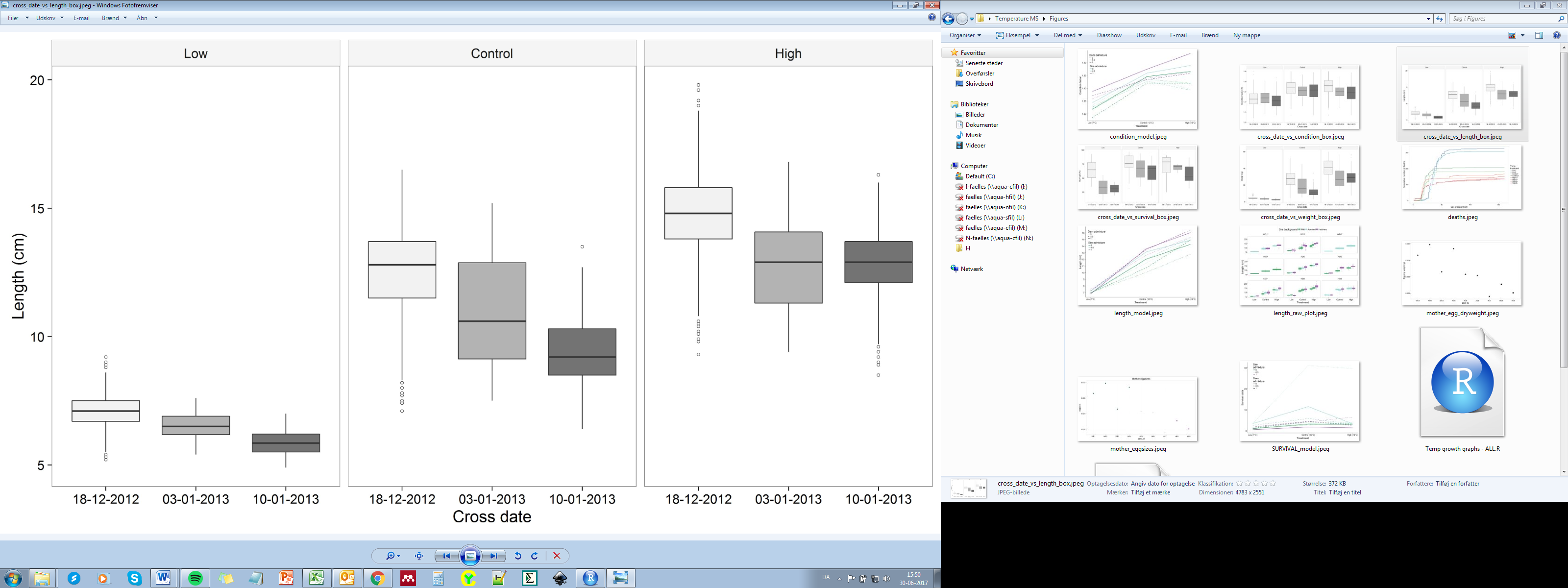


Figure S2. The effect of cross date on lengths of juvenile trout reared under three temperature regimes (Low = 7 ˚C, Control = 12 ˚C and High = 16 ˚C). Boxes indicate 1^st^, 2^nd^ (median) and 3^rd^ quartiles. Upper whisker = min(max(x), Q_3 + 1.5 * IQR), lower whisker= max(min(x), Q_1 - 1.5 * IQR), and dots indicate outliers.


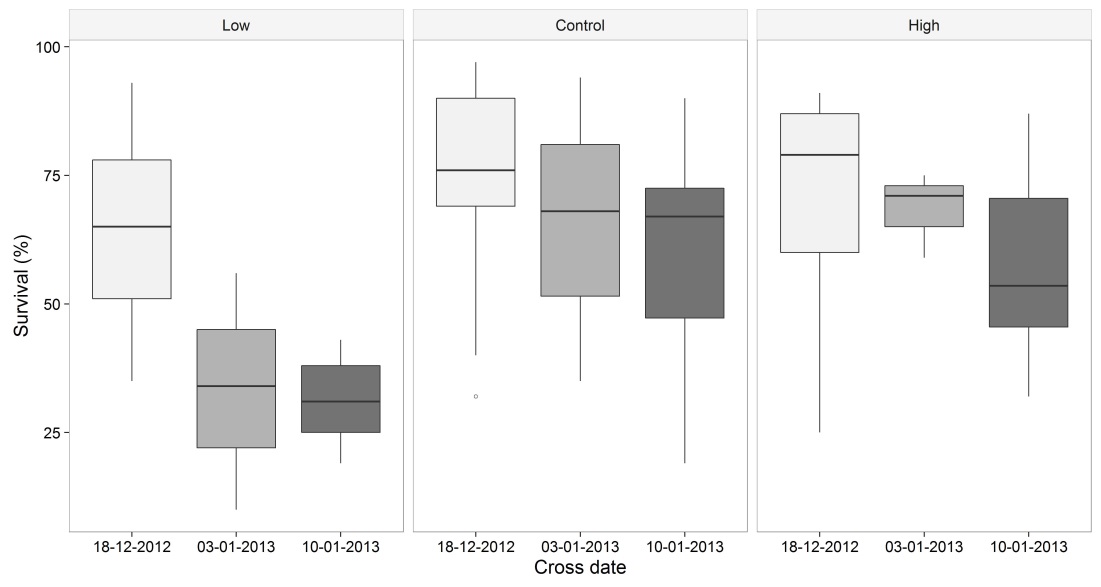


Figure S3. The effect of cross date on the survival of juvenile trout reared under three temperature regimes (Low = 7 ˚C, Control = 12 ˚C and High = 16 ˚C). Boxes indicate 1^st^, 2^nd^ (median) and 3^rd^ quartiles. Upper whisker = min(max(x), Q_3 + 1.5 * IQR), lower whisker= max(min(x), Q_1 - 1.5 * IQR), and dots indicate outliers.
